# Antibacterial and Fluorescence Staining Properties of an Innovative GTR Membrane Containing 45S5BGs and AIE Molecules In Vitro

**DOI:** 10.3390/nano12040641

**Published:** 2022-02-14

**Authors:** Yu-Wen Wei, Sayed Mir Sayed, Wei-Wen Zhu, Ke-Fei Xu, Fu-Gen Wu, Jing Xu, He-Peng Nie, Yu-Li Wang, Xiao-Lin Lu, Qian Ma

**Affiliations:** 1 Jiangsu Province Key Laboratory of Oral Diseases, Department of General Dentistry, The Affiliated Stomatological Hospital of Nanjing Medical University, Nanjing Medical University, Nanjing 210029, China; yww0425@163.com (Y.-W.W.); weiwenzhu@njmu.edu.cn (W.-W.Z.); dentistxj@sina.com (J.X.); the3ears@163.com (H.-P.N.); njykdwyl@njmu.edu.cn (Y.-L.W.); 2 State Key Laboratory of Bioelectronics, School of Biological Science and Medical Engineering, Southeast University, 2 Sipailou Road, Nanjing 210096, China; 101101849@seu.edu.cn (S.M.S.); kf.xu@seu.edu.cn (K.-F.X.); wufg@seu.edu.cn (F.-G.W.)

**Keywords:** aggregation-induced emission nanoparticles, antibacterial, antibacterial differentiation, bioactive glass, bioprobe, guided tissue regeneration (GTR)

## Abstract

This study aimed to add two functional components—antibacterial 45S5BGs particles and AIE nanoparticles (TPE-NIM^+^) with bioprobe characteristics—to the guided tissue regeneration (GTR) membrane, to optimize the performance. The PLGA/BG/TPE-NIM^+^ membrane was synthesized. The static water contact angle, morphologies, and surface element analysis of the membrane were then characterized. In vitro biocompatibility was tested with MC3T3-E1 cells using CCK-8 assay, and antibacterial property was evaluated with *Streptococcus mutans* and *Porphyromonas gingivalis* by the LIVE/DEAD bacterial staining and dilution plating procedure. The fluorescence staining of bacteria was observed by Laser Scanning Confocal Microscope. The results showed that the average water contact angle was 46°. In the cytotoxicity test, except for the positive control group, there was no significant difference among the groups (*p* > 0.05). The antibacterial effect in the PLGA/BG/TPE-NIM^+^ group was significantly (*p* < 0.01), while the sterilization rate was 99.99%, better than that in the PLGA/BG group (98.62%) (*p* < 0.01). Confocal images showed that the membrane efficiently distinguished G^+^ bacteria from G^−^ bacteria. This study demonstrated that the PLGA/BG/TPE-NIM^+^ membrane showed good biocompatibility, efficient sterilization performance, and surface mineralization ability and could be used to detect pathogens in a simple, fast, and wash-free protocol.

## 1. Introduction

Periodontitis is an inflammatory disease affecting the periodontal tissues, including alveolar bone, periodontal ligament, cementum, and other dental supporting tissues [1]. Peri-implantitis is a pathological condition occurring in tissues around dental implants, which is characterized by inflammation in the peri-implant connective tissue and progressive loss of the supporting bone [2]. People suffering from periodontitis (or peri-implant inflammation) are more likely to lose their teeth or implants [1,2]. Thus, patients with severe periodontal defects caused by periodontitis require surgical treatment. However, if the periodontal defects are left empty after the debridement, epithelial cells and fibroblasts first fill the defect area and then form fibro-epithelial tissues, preventing further regeneration of the periodontal tissue. In this regard, the guided tissue regeneration (GTR) technology should be used clinically to prevent epithelial cells, fibers, and gingival connective tissue through a physical barrier. Creating an isolated space for the introduction of blood vessels and bone progenitor cells can prevent the growth of non-osteoblastic tissues [1]. The environment of the oral cavity is rich in bacteria. Therefore, post GTR surgery infection is one of the most common complications, which often leads to the poor prognosis of GTR surgeries [3]. It is difficult to load antibiotic on the GTR membranes, therefore, the infections are usually treated with a short-term antibiotic [4,5]. However, bacteria tend to develop resistance towards antibiotics, which makes therapeutic effect of antibiotics controversial [6,7]. Therefore, the use of antibiotics is not the best choice for the complication treatment of GTR surgeries. Herein, we report the development of a novel GTR membrane, which has feasible antibacterial property without drug resistance.

For this purpose, 45S5BGs, a classic type of bioactive glass, was chosen for its antibacterial activity [8,9], healing ability to soft tissues [10,11,12], remineralization characteristics [13], and adhesion and proliferation properties for osteoblasts [14,15,16] in this study. 45S5BGs can be used to treat bacteria-related oral diseases against a variety of anaerobic bacteria, non-anaerobic bacteria, and other common clinical microorganisms. Many needle-like fragments on the surface of 45S5BGs and the area close to the cell walls are observed under a transmission electron microscope (TEM) [8]. These structures can pierce the bacterial cell walls and force the bacteria to lose their intact structures. In addition, 45S5BGs can release Na^+^, Ca^2+^, and PO_4_^3−^ ions in a physiological environment, resulting an increase in the pH [8,17,18]. The alkaline environment can thus reduce the surface colonization of bacteria in the body [19], cause a certain pressure on the bacteria, and change their shape to adapt to the changes in the environment. Specifically, the integrity of the cytoplasmic membrane of the bacteria is destroyed with current protein denaturation, abnormal protein expression DNA damage, and malfunction of enzyme metabolism, which can finally result in the bacterial death [20]. Our previous study [21] showed that 45S5BGs had a high antibacterial activity against oral bacteria including *Streptococcus sanguis* plaques and mixed plaques. 45S5BGs particles were added to the poly(lactide-*co*-glycolide) (PLGA) gel dispersion to endow the membrane with antibacterial activity. Additionally, compared with the antibiotics, 45S5BGs offer the advantage for the oral bacteria not to develop resistance against the antibacterial property [22]. Therefore, applying 45S5BGs particles to the PLGA gel dispersion can provide the membrane with the antibacterial activity, remineralization characteristics, and adhesion and proliferation properties for osteoblasts.

**Scheme 1:**
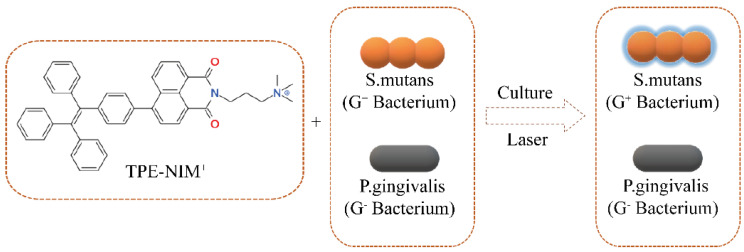
Molecular structures of TPE-NIM^+^ and a general representation of their adsorption onto the surfaces of G^+^ bacterium. Note that the TPE-NIM^+^ and the bacteria are not drawn in real scales.

Furthermore, previous studies showed that the most common complications after the GTR surgery were mixed oral bacterial infections [23]. For instance, the numbers of G^+^ aerobic bacteria increased in the initial stage of infection, and then G^−^ aerobic bacteria provided favorable conditions for further adhesion of G^+^ and G^−^ anaerobic bacteria [24]. Considering the threat posed by pathogens, their fast discrimination and identification are highly demanded to help the clinicians in targeted infection control. For this purpose, in previous studies [25,26], we synthesized a naphthalimide-based bioprobe named tetraphenylethylene-naphthalimide (TPE-NIM) with aggregation-induced emission (AIE) characteristic [25] and then modified it to prepare TPE-NIM^+^ [26]. In 2016, AIE nanoparticles were listed as one of the four major nanomaterials supporting the “Nano-optical Revolution” by *Nature*. In recent years, fluorescence imaging technology has attracted widespread attention in the biomedical community due to its simple usability, high selectivity and sensitivity to biological analytes, good brightness, and light stability, which can be used in the real-time monitoring of complex biological processes [27,28]. However, conventional fluorescent probes, such as rhodamine, fluorescein, BODIPY, and anthocyanins, are affected by the aggregation quenching and severe photobleaching properties in the long-term imaging processes, which greatly limit their practical clinical applications. To resolve such issues, we have synthesized a naphthalimide-based bioprobe, named tetraphenylethylene-naphthalimide (TPE-NIM) [25] which was further modified into TPE-NIM^+^ [26] with the AIE characteristic. The AIE behavior of TPE-NIM was studied in dimethyl sulfoxide (DMSO)/water system. The size of the TPE-NIM nanoaggregates were related to the water content. The size was decreased from 51.2 ± 15.6 nm at 70% water fraction to 27.2 ± 6.8 nm at 90% water fraction. The AIE behavior of TPE-NIM^+^ and TPE-NIM are almost similar. These two multifunctional AIEgens (TPE-NIM and TPE-NIM^+^) could track and identify G^+^ bacteria with fast stainability, high selectivity, and wash-free property [27]. Specifically, TPE-NIM^+^, just like TPE-NIM, showed a high degree of binding/imaging selectivity for G^+^ bacteria over G^−^ bacteria and fungi via a wash-free protocol, as shown in Scheme 1. We have also confirmed that the binding mechanism of TPE-NIM^+^ and G^+^ bacteria was mainly the electrostatic attraction/interaction between the positively charged amine and the negatively charged plasma membrane and the hydrophobic interaction between AIEgen and the hydrophobic lipid tail [26]. The tertiary amine, as the biologically active component of the designed AIEgens, could strongly interact with the lipoteichoic acid and teichuronic acid present in the cell wall of G^+^ bacteria via electrostatic interaction and might play a key role in the selective staining of the pathogens [29]. For this purpose, we innovatively introduced TPE-NIM^+^ into the composite membrane as a selective fluorescent nano bioprobe. As the membrane degraded in vivo, TPE-NIM^+^ could be released from the membrane into the body fluid such as gingival crevicular fluid, pus, or blood, which was collected and examined to determine whether the patients’ periodontitis was primarily caused by G^+^ or G^−^ bacteria. Identification of bacterial species might help clinicians better evaluate the postoperative status and provide further treatment to the periodontal affected patients.

In addition, poly(lactide-*co*-glycolide) (PLGA) is a kind of biodegradable polymer that has been widely investigated as a material for GTR. In this study, PLGA was adopted as the matrix under physiological conditions because of its advantages of safety, reasonable degradation rate, and easy availability [30,31,32]. PLGA/BG and PLGA/BG/TPE-NIM^+^ composite membranes were then prepared. The morphologies and surface element analysis of the membranes were characterized by using scanning electron microscopy–energy-dispersive spectrometry (SEM–EDS). The composite membrane was immersed in simulated body fluid (SBF) to examine its apatite-forming ability. SBF is a simple solution without a complex physiological environment of the cells and proteins; it has an ion composition similar to that of human plasma. Whether the SBF immersion test can correctly predict the biological activity in vivo is debatable, but this method is still widely used to reflect the remineralization characteristics of materials [13,33]. The in vitro biocompatibility of the membranes was tested with MC3T3-E1 cells using CCK-8 assay. The results indicated that composite membranes were almost noncytotoxic. The in vitro antibacterial property of the membranes was evaluated with *Streptococcus mutans* and *Porphyromonas gingivalis* by the LIVE/DEAD bacterial staining and dilution plating procedure. This novel membrane showed significant antibacterial activity, G^+^ bacterial stainability, and remineralization properties.

## 2. Materials and Methods

### 2.1. Materials and Instruments

**Scheme 2:**
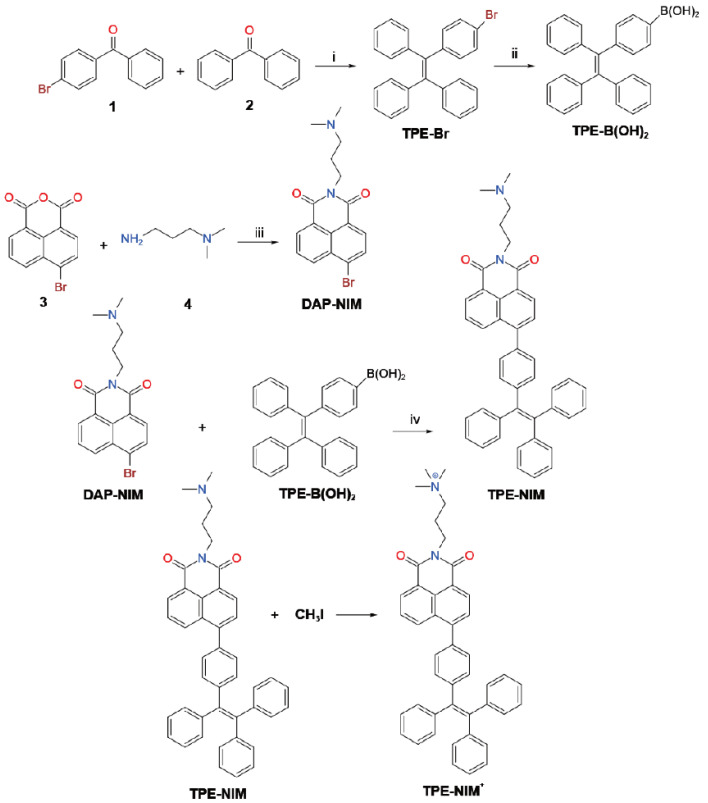
Synthetic routes of TPE-NIM^+^. Reagents and conditions: (i) Zn, TiCl_4_, tetrahydrofuran (THF), 0–25 °C, 12 h, (ii) n-Butyllithium solution, trimethyl borate, THF, –78 °C, 3 h, (iii) C_2_H_5_OH, reflux, 2 h, and (iv) Pd(PPh_3_)_4_, Na_2_CO_3_, mixed solvent (toluene: ethanol: water), 90 °C, 12 h.

All chemicals used for the synthesis were reagent grade and were used without further purification. Poly(lactide-*co*-glycolide) (PLGA), (LA/GA = 75:25, Mw = 7000–17,000) was purchased from Shanghai Yuanye Bio-Technology Co., Ltd. (Shanghai, China). The 45S5BGs particles (Actimins) were purchased from Datsing Bio-tech Co., Ltd. (Beijing, China), with the following weight composition: 45 SiO_2_, 24.5 CaO, 24.5 Na_2_O, and 6.05 P_2_O_5_. The particle size of most 45S5BGs is approximately in the range of 1–20 µm, and 5% of the particles are less than 1 µm in diameter. Tetraphenylethylene-naphthalimide^+^ (TPE-NIM^+^) was designed and synthesized as described in our previous studies [25,26,34]. The synthesis routes were shown in Scheme 2. The chemical structure was then characterized using proton nuclear magnetic resonance (NMR) (Jeol JNM-ECZS 400 MHz, Akishima, Japan) in DMSO-d6 at room temperature. The Millipore filtration system (Billerica, MA, USA) was used for water purification. *P. gingivalis* (ATCC 33277) and *S. mutans* (ATCC 25175) strains were obtained from Shanghai Bioresource Collection Center (Shanghai, China).

### 2.2. Synthesis of PLGA/BG and PLGA/BG/TPE-NIM^+^ Membranes

PLGA was first dissolved in tetrahydrofuran (THF, Aladdin, Shanghai, China) with sonication. TPE-NIM^+^ and 45S5BGs were then sequentially added and sonicated into the solvent. The solution was evaporated in a Teflon container under the nitrogen flow to get a film. The resulting film was named as PLGA/45S5BGs/TPE-NIM^+^ (abbreviated as PLGA/BG/TPE-NIM^+^). The film without adding TPE-NIM^+^ was called PLGA/45S5BGs (abbreviated as PLGA/BG).

### 2.3. Characterization of PLGA/BG and PLGA/BG/TPE-NIM^+^ Membranes

#### 2.3.1. Water Contact Angle Measurement of the PLGA/BG/TPE-NIM^+^ Membrane

The static water contact angles were measured to characterize the hydrophilicity of the membranes. The contact angle measuring instrument (JC2000C1, Shanghai, China) was purchased from Shanghai Zhongchen Digital Technic Apparatus Co., Ltd. A distilled water droplet (20 µL) was released from a syringe and dropped on a prepared membrane. Three measurements were done at different positions for each sample, and the results were the averaged ones.

#### 2.3.2. Scanning Electron Microscopy/Energy-Dispersive Spectrometry

The morphological information of PLGA/BG/TPE-NIM^+^ membranes, before and after the immersion in the SBF (1× SBF) in a shaker at 37 °C (200 rpm), was obtained using scanning electron microscopy/energy-dispersive spectrometry (SEM/EDS, LEO 1530VP, ZEISS, Oberkochen, Germany). The micrographs were acquired using beam energy of 5.0–20.0 kV and a working distance of 4.83–5.28 mm. Before SEM/EDS, the membranes were sequentially fixed using a glutaraldehyde (Solarbio, Beijing, China) solution (2.5 wt%), stored overnight at 4 °C, rinsed with 1× phosphate-buffered saline (PBS), gradient-dehydrated with ethanol, dried, and sputter-coated with platinum.

### 2.4. Cell Culture

MC3T3-E1 cells were cultured in MEMα (Gibco, Carlsbad, CA, USA) supplemented with 10% fetal bovine serum (Gibco). The medium was replaced every three days, and the cultures were maintained in a humidified 5% CO_2_ incubator at 37 °C.

### 2.5. Cytotoxicity Test

The PLGA/BG/TPE-NIM^+^ composite membranes containing 10 wt%, 20 wt%, and 30 wt% 45S5BGs were prepared. The CCK-8 test was performed for cytotoxicity. PLGA/10%BG/4%TPE-NIM^+^, PLGA/20%BG/4%TPE-NIM^+^, and PLGA/30%BG/4%TPE-NIM^+^ sample flakes with the surface size of 5 × 5 mm^2^ were cut from the original films for the cytotoxicity test. MC3T3-E1 cells were seeded at a concentration of 1 × 10^5^/mL and 37 °C using 96-well plates (100 μL of MEMα/well) in a humidified atmosphere with 5% CO_2_ for 24 h. Four groups were set: (i) cells only (negative control), (ii) cells with PLGA/10%BG/4%TPE-NIM^+^ flake, (iii) cells with PLGA/20%BG/4%TPE-NIM^+^ flake; and (iv) cells with PLGA/30%BG/4%TPE-NIM^+^ flake. Two parallel samples were prepared for each group. After incubation for one, two, three, four, and five days, the culture medium was extracted, 10% (*v*/*v*) CCK-8 (Beyotime, Shanghai, China) was added, and the plates were incubated in the dark for 2 h. The optical density (OD) value of each sample was then determined using an ultraviolet spectrophotometer (SpectraMax, Molecular Devices, San Jose, CA, USA) at 450 nm. The result showed that the composite membranes containing 20% and 30% 45S5BGs had certain cytotoxicity (*p* < 0.05). Therefore, we finally chose composite membranes containing 10% 45S5BGs for the future experiment.

PLGA/10%BG and PLGA/10%BG/4%TPE-NIM^+^ sample flakes with a size of 5 × 5 mm^2^ were cut from the original processed films for the cytotoxicity tests. MC3T3-E1 cells were seeded into 96-well plates at a concentration of 1 × 10^5^/mL (100 µL of MEMα/well) at 37 °C in a humidified atmosphere with 5% CO_2_. After 24 h, when all cells had attached, the sterilized samples were placed into 96-well plates. Five groups were set: (i) no cells (blank control), (ii) cells with 6.3% phenol (positive control), (iii) cells only (negative control), (iv) cells with PLGA/BG flake (PLGA/BG group), and (v) cells with PLGA/BG/TPE-NIM^+^ flake (PLGA/BG/TPE-NIM^+^ group). Three parallel samples were prepared for each group. After incubation for one, two, three, four, and five days, the old medium was extracted, 10% (*v*/*v*) CCK-8 (Beyotime, Shanghai, China) was added, and the plates were incubated in the dark for 2 h. The OD value of each sample was then determined using an ultraviolet spectrophotometer (SpectraMax, Molecular Devices) at 450 nm. Cell viability rate was calculated according to the OD value of five days and the cell viability rate after 48 h of treatment by formula (1):

(1) $$\mathrm{Cell}\;\mathrm{Viability}\;(\%)=\frac{As-Ab}{Ac-Ab}\times 100\%,$$

*As*: Absorbance value of test groups

*Ac*: Absorbance value of negative control group

*Ab*: Absorbance value of blank control group

### 2.6. Bacterial Culture

*P. gingivalis* (ATCC 33277) and *S. mutans* (ATCC 25175) were resuscitated for 48 h and then inoculated in brain heart infusion (BHI, Solarbio) liquid medium under anaerobic conditions (5% CO_2_, 10% H_2_, and 85% N_2_) for later use.

### 2.7. Antibacterial Activity Test

Different sample flakes were prepared into 10 × 5 mm^2^ square flakes and irradiated with an ultraviolet lamp for 30 min to ensure sterility. Four groups were set: (i) bacterial solution only (blank control), (ii) bacterial solution with PLGA flake (negative control), (iii) bacterial solution with PLGA/BG flake (PLGA/BG group), and (iv) bacterial solution with PLGA/BG/TPE-NIM^+^ flake (PLGA/BG/TPE-NIM^+^ group). Then, 1 mL of BHI liquid medium was added to all the tubes and incubated in a constant-temperature shaker (37 °C, 200 rpm) for 48 h to dissolve the components in flakes. *S. mutans* was cultured on BHI solid medium and harvested in the exponential growth phase via centrifugation. After the bacteria were resuspended in BHI liquid medium, the concentration of the bacteria solution was adjusted to 2 × 10^3^ colony-forming units (CFUs)/mL using a bacterial turbidimeter (Xinrui Instruments, WGZ-2XJ, Shanghai, China). Then, 1 mL of the prepared bacteria solution was added to all the tubes. The volume of the co-cultivation system was 2 mL, cultured under anaerobic conditions (5% CO_2_, 10% H_2_, and 85% N_2_) for 24 h. For the negative control and PLGA/BG/TPE-NIM^+^ groups, a LIVE/DEAD BacLight Bacterial Viability Kit for microscopy and quantitative assays (Invitrogen, Carlsbad, CA, USA) was used for qualitative analysis under an upright fluorescence microscope (Leica, DM4000, Wetzlar, Germany). The quantitative analysis was performed on all groups: the bacterial solution was diluted with different concentrations and spread on the plate. After 24 h, the number of colonies formed in each plate was recorded. The bacterial solutions were diluted at different concentrations and spread on the plates. The number of colonies formed in each plate was recorded. The total numbers of bacterial colonies contained in the original samples were calculated based on the dilution factor. Each experiment was performed in triplicate.

(2) $$\mathrm{Sterilizing}\;\mathrm{rate}\;(\%)=\frac{A-B}{A}\times 100\%,$$

*A* and *B* correspond to the numbers of CFU/mL in the blank and experimental groups, respectively.

### 2.8. Staining Abilities of TPE-NIM^+^ toward Pathogens

The front and back sides of the membrane were irradiated under an ultraviolet lamp for 30 min. PBS was used as the extraction medium, the membrane was leached under anaerobic conditions (5% CO_2_, 10% H_2_, and 85% N_2_) for 24 h at a ratio of 1 cm^2^/mL, the extract was then taken, and a bacterial filter was used to filter out 45S5BGs particles. *S. mutans* (G^+^) and *P. gingivalis* (G^−^) were selected to study the combined effect of the designed TPE-NIM^+^ on the common pathogens of periodontal diseases. *S. mutans* and *P. gingivalis* were incubated with the extract for 15 min at 37 °C, and the staining performance of TPE-NIM^+^ on the two bacteria was checked using a confocal microscope. In addition, we also performed staining experiments on mixed G^+^ bacteria (*S. m*) and G^−^ bacteria (*P. g*) (1:1). The bacterial mixture was incubated with the extract for 15 min, and the results of the staining were observed under a confocal microscope. We kept 45S5BGs particles in the membrane extract without using a bacterial filter, and *S. mutans* and *P. gingivalis* were incubated with the extract for 15 min at 37 °C to study the effect of 45S5BGs on bacteria and the staining effect of TPE-NIM^+^ on dead bacteria. The staining performance of TPE-NIM^+^ on dead bacteria was observed under a confocal microscope.

### 2.9. Statistical Analysis

The experimental data were expressed as mean ± standard deviation. The statistical analysis for significance between the groups was performed using the Student *t* test and one-way analysis with SPSS25.0 software. A confidence level of 95% (*p* < 0.05) was statistically significant. Each experiment was done in triplicate.

## 3. Results

### 3.1. Synthesis and Characterization of TPE-NIM^+^

**Figure 1:**
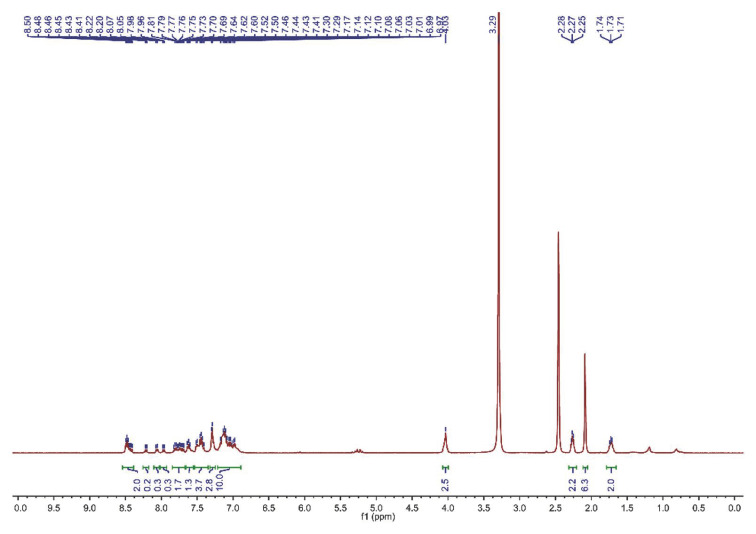
The Proton NMR of TPE-NIM^+^.

TPE-NIM^+^, as a derivative of TPE-NIM, was synthesized via a similar approach with a further modification step; the synthetic procedures of TPE-NIM^+^ have been reported in our previous studies [25,26]. The resulting TPE-NIM^+^ was obtained as a dark yellow solid (yield: B60%). The chemical structure of TPE-NIM^+^ was confirmed by Proton-NMR: 1H-NMR (400 MHz, DMSO-d6) δ 8.41–8.50 (m, 2H), 7.96–8.22 (m, 1H), 7.69–7.83 (m, 2H), 7.57–7.64 (m, 1H), 7.41–7.52 (m, 3H), 7.27–7.30 (m, 3H), 6.97–7.22 (m, 10H), 4.03 (t, J = 4.4 Hz, 2H), 2.27 (t, J = 6.8 Hz, 2H), 2.08 (d, J = 4.0 Hz, 6H), and 1.80–1.63 (m, 2H) (Figure 1).

### 3.2. Preparation of PLGA/BG and PLGA/BG/TPE-NIM^+^ Membranes

Novel poly(lactide-*co*-glycolide)/45S5BGs (hereinafter called PLGA/BG) and poly(lactide-*co*-glycolide)/45S5BGs/TPE-NIM^+^ (hereinafter called PLGA/BG/TPE-NIM^+^) composite membranes were produced using a chemical synthesis method described previously. The PLGA/BG membrane with a weight ratio of 9:1 was named PLGA/10%BG membrane, and the one with an extra 4% TPE-NIM^+^ content was named PLGA/10%BG/4%TPE-NIM^+^ membrane. The membranes containing 20% and 30% BG were not used for the further test; therefore, no detailed explanation was given.

### 3.3. Characterization of PLGA/BG and PLGA/BG/TPE-NIM^+^ Membranes

#### 3.3.1. Water Contact Angle Measurement of the PLGA/BG/TPE-NIM^+^ Membrane

**Figure 2:**
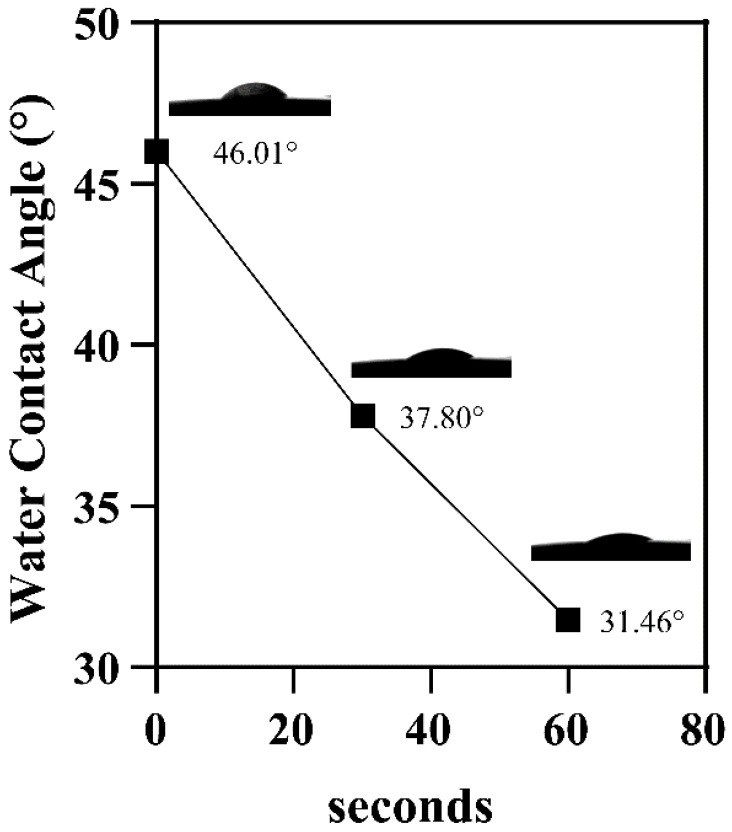
Images of the surface of the PLGA/BG/TPE-NIM^+^ membrane after putting a drop of distilled water on it. Measured water contact angle values and captured images at 0 s, 30 s, and 60 s.

Figure 2 shows the static water contact angle test results of the PLGA/BG/TPE-NIM^+^ film. When distilled water was just dropped onto the surface of the film, the average water contact angle was 46°. After 30 and 60 s, the contact angle gradually decreased. After about 120 s, the contact angle was nearly 0°, at which time the distilled water droplets were completely infiltrated. The PLGA/BG/TPE-NIM^+^ membrane showed moderate hydrophilicity. The hydrophilicity of biomaterials had an important impact on the adhesion and proliferation of cells on the material. The surface of biomaterials with moderate hydrophilicity could promote cell growth and improve biocompatibility [35].

#### 3.3.2. Scanning Electron Microscopy/Energy-Dispersive Spectrometry

**Figure 3:**
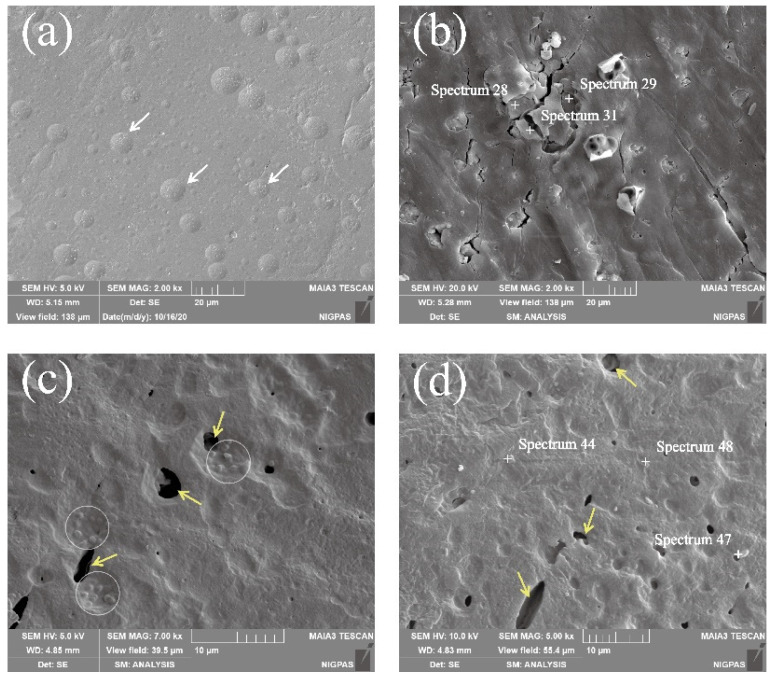
SEM images of composite membranes before and after immersion in SBF. (**a**,**b**) SEM images of PLGA/10%BG/4%TPE-NIM^+^ membrane before immersion in SBF. White arrows show the exposed 45S5BGs particles. (**b**) Three fixed points (Spectrum 28.29.31) were selected randomly for EDS analysis. (**c**,**d**) SEM images of PLGA/10%BG/4%TPE-NIM^+^ membrane after immersion in SBF for three days: (**c**) White circles showed the embedded 45S5BGs particles. Yellow arrows showed the hollow structure; and (**d**) Yellow arrows show the hollow structure. Three fixed points (Spectrum 44.47.48) were selected randomly for EDS analysis.

**Figure 4:**
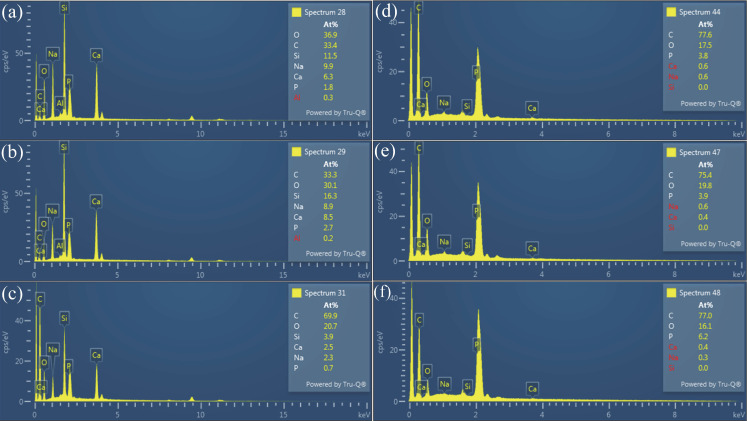
EDS analysis of composite membranes before and after immersion. (**a**–**c**) EDS images of PLGA/10%BG/4%TPE-NIM^+^ membrane without immersion in SBF. (**d**–**f**) EDS images of PLGA/10%BG/4%TPE-NIM^+^ membrane after three days of immersion in SBF.

**Figure 5:**
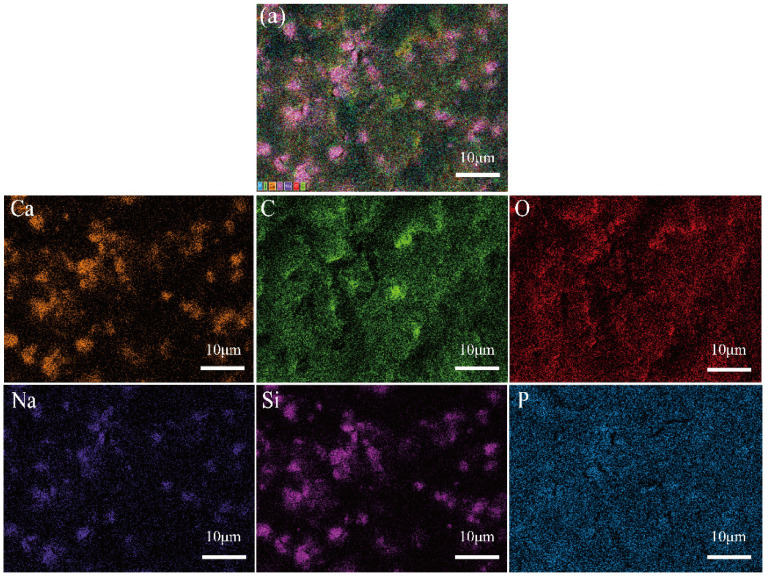
EDS Elemental Analysis with ColorSEM Technology of the PLGA/10%BG/4%TPE-NIM^+^ membrane without immersion in SBF. (**a**) EDS image with all elements superimposed.

The microstructure and morphology of membranes of the PLGA were observed using an SEM. The representative SEM images of PLGA/10%BG/4%TPE-NIM^+^ membranes (before and after immersion in SBF) are displayed in Figure 3. As shown in Figure 3a,b, small spherical protrusions (about 5–10 µm in diameter) were regularly arranged on membrane surfaces without immersion in SBF. Also, EDS images (Figure 4a–c) showed that the Ca/P ratio was approximately 4:1, which was consistent with the composition ratio of 45S5BGs particles, indicating that the spherical protrusions on the membrane surface were 45S5BGs particles embedded in the composite membrane. EDS elemental analysis using ColorSEM technology reached the same conclusion (Figure 5), calcium, sodium, and silicon elements aggregated where 45S5BGs particles were located.

**Figure 6:**
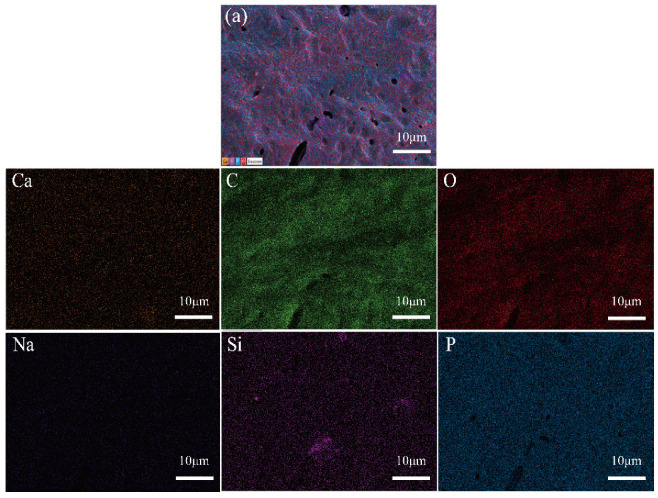
EDS Elemental Analysis with ColorSEM Technology of the PLGA/10%BG/4%TPE-NIM^+^ membrane after three days of immersion in SBF. (**a**) EDS image with all elements superimposed.

After the membrane was immersed in SBF for three days, the acidic environment formed by PLGA degradation further accelerated its own degradation. Consequently, the degradation rate inside the material was greater than that on the surface, finally forming a hollow structure (Figure 3c,d). Meanwhile, embedded 45S5BGs particles were exposed, as shown in Figure 3c. EDS images (Figure 4d,f) showed that phosphorus elements rise, calcium content fell, and no silicon element was detected on the surface of the membrane. 45S5BGs wrapped in the membrane were dissolved, and calcium and phosphorus were released from the film, which was different from the concentrated distribution before soaking, but were evenly dispersed on the surface of the membrane (Figure 6). Calcium and phosphorus are two main elements of mineralization, and the uniform distribution on the surface of the film will provide conditions for the formation of subsequent apatite-like structures.

### 3.4. Cytotoxicity Assay

**Figure 7:**
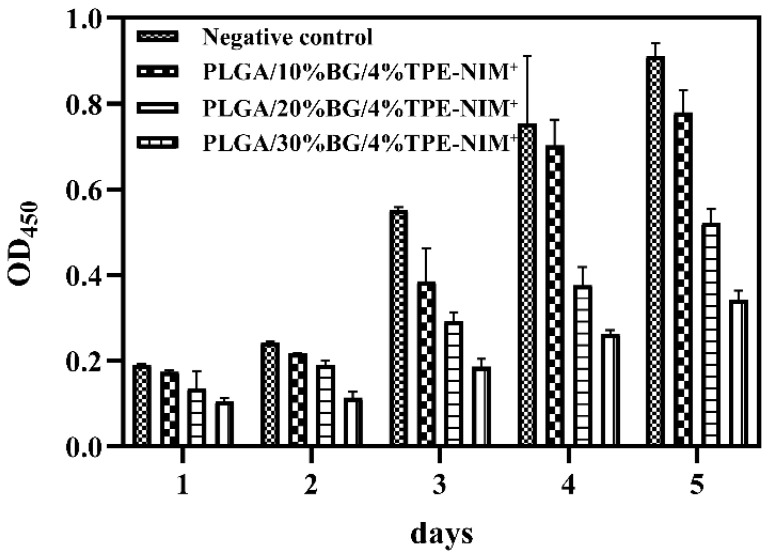
Cytotoxicity of control medium, PLGA/BG/TPE-NIM^+^ composite membranes, respectively, containing 10%, 20%, or 30% BG toward MC3T3-E1.

**Figure 8:**
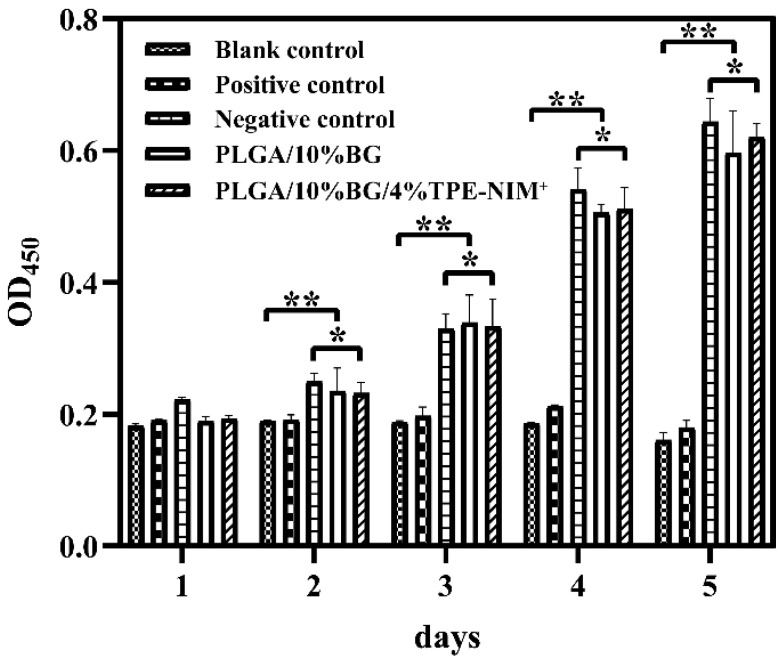
Cytotoxicity of control medium, PLGA/BG membrane and PLGA/BG/TPE-NIM^+^ membrane toward MC3T3-E1. (* *p* > 0.05 ** *p* < 0.05 vs. blank control group).

The CCK-8 method was applied to detect the viability rate of MC3T3-E1 cells. As shown in Figure 7, the results of the previous experiment showed that the viability rate of cells co-cultured with membranes containing 10% 45S5BGs was more than 70%, which was much better than that for membranes containing 20% or 30% 45S5BGs. Therefore, we only selected membranes containing 10% 45S5BGs in the further test. As shown in Figure 8, the OD of each group increased after five days of co-cultivation. No significant difference was observed between the blank and positive control groups (*p* > 0.05); therefore, false negatives were excluded. Two days after co-cultivation, no significant difference was noted between all groups. After three days, the negative control group showed significant differences compared with the blank control group (*p* < 0.05). No significant difference was found between the negative control, PLGA/BG group, and PLGA/BG/TPE-NIM^+^ group (*p* > 0.05). It showed that neither 45S5BGs nor TPE-NIM^+^ showed obvious cytotoxicity. After 48 h of treatment with the PLGA/BG membrane and PLGA/BG/TPE-NIM^+^ membrane, the cell viability rate was 75.1% and 70.2%, respectively. According to the ISO 10993-5 standard, the cell viability rate was greater than 70%. Therefore, both PLGA/BG and PLGA/BG/TPE-NIM^+^ membranes could be considered as noncytotoxic.

### 3.5. Antibacterial Activity Test

*S. mutans* colony formed on the solid medium was observed. The size was about 1–2 mm, the shape was a white circle with neat edges, and the appearance was bright, smooth, moist, and viscous. The total numbers of bacterial colonies contained in the original samples were calculated according to the dilution factor. The statistical results showed that the number of colonies in the blank control, negative control, and PLGA/BG group, and PLGA/BG/TPE-NIM groups was 8.37 × 10^13^ CFU/mL, 9.73 × 10^13^ CFU/mL, 1.17 × 10^12^ CFU/mL, and 4.03×10^9^ CFU/mL, respectively. The statistical results showed no significant difference between the blank control and negative control groups (*p* > 0.05), which verified that the PLGA component had no significant antibacterial properties. Compared with the negative control group, the antibacterial effect in the PLGA/BG group was significantly different (*p* < 0.01): the sterilization rate was 98.62%, which showed that 45S5BGs had excellent antibacterial properties. In addition, the sterilization rate in the PLGA/BG/TPE-NIM^+^ group was 99.99%, which showed better sterilization performance than that in the PLGA/BG group (*p* < 0.01). Under the experimental culture conditions, the surface of the bacteria was negatively charged, while that of TPE-NIM^+^ was positively charged. Hence, electrostatic attraction/interaction existed between the positively charged amine and the negatively charged plasma membrane. Also, a hydrophobic interaction was noted between TPE-NIM^+^ and the hydrophobic lipid tail [26]. These interactions might inhibit the respiration of bacteria or change the number of surface electric charges, which might be the reason why TPE-NIM^+^ had antibacterial properties.

**Figure 9:**
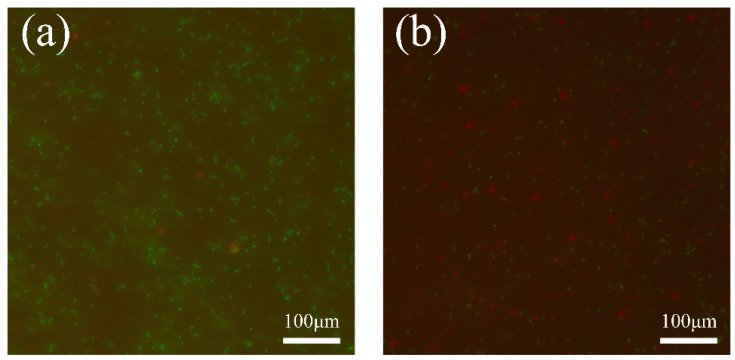
Live and Dead bacterial staining. (**a**) *S. m* bacteria liquid without the treatment with PLGA/BG/TPE-NIM^+^ composite membrane. (**b**) *S. m* bacteria liquid with the treatment with PLGA/BG/TPE-NIM^+^ composite membrane for 24 h.

An upright fluorescence microscope image (Figure 9) showed that many live bacteria that emitted green fluorescence in the bacterial solution without membrane treatment (Figure 9a) were swimming, showing good viability. Compared with the PLGA/BG/TPE-NIM^+^ membrane-treated bacterial solution (Figure 9b), the proportion of live bacteria that emitted green fluorescence was greatly reduced, while the proportion of dead bacteria that emitted red fluorescence increased. The qualitative test assisted in proving that the membrane we prepared had antibacterial properties.

### 3.6. Staining Abilities of TPE-NIM^+^ toward Pathogens

**Figure 10:**
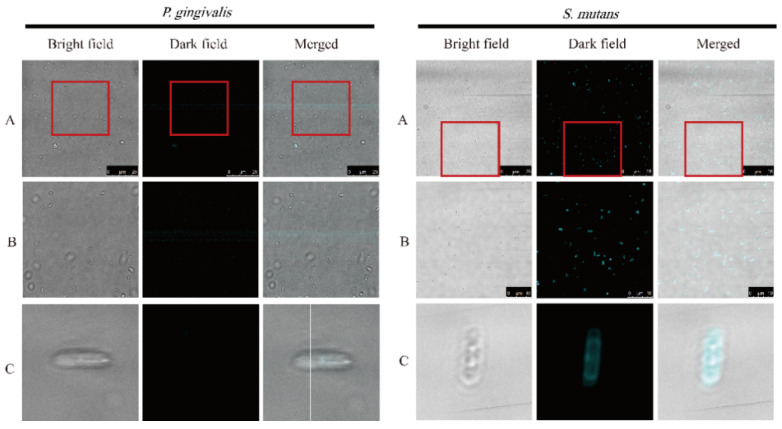
Confocal images of *P. gingivalis* (**left**: **A**–**C**) and *S. mutans* (**right**: **A**–**C**) after treatment with PLGA/10%BG/4%TPE-NIM^+^ membrane extract (without 45S5BGs) for 15 min.

**Figure 11:**
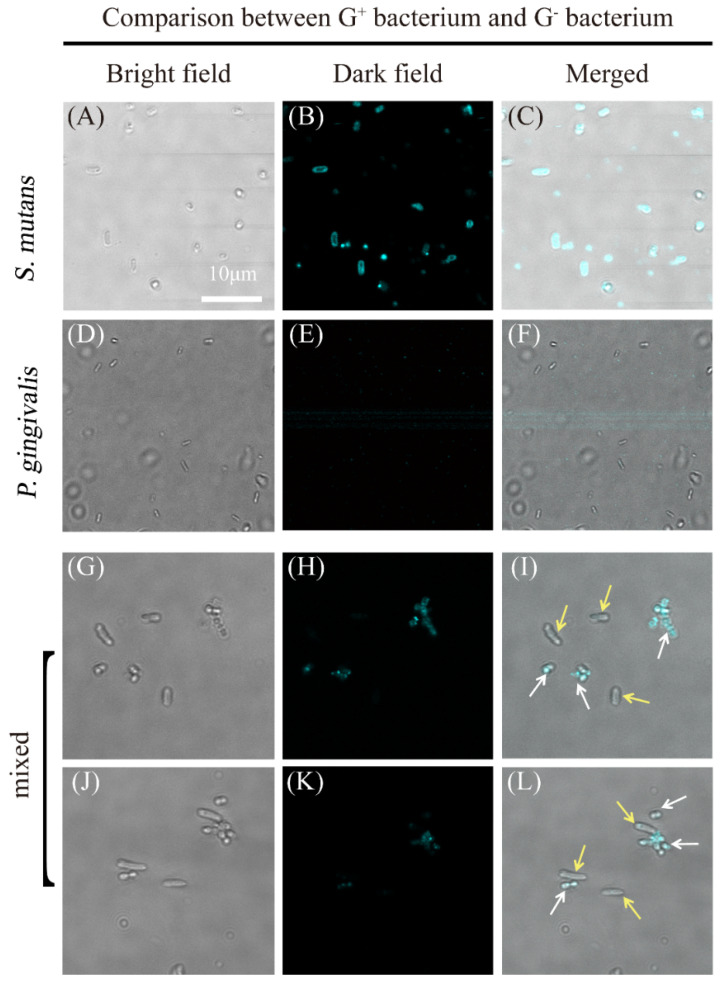
Confocal images of *S. mutans* (**A**–**C**) and *P. gingivalis* (**D**–**F**) after treatment with PLGA/10%BG/4%TPE-NIM^+^ membrane extract (without 45S5BGs) for 15 min. (**G**–**L**) Selective staining of *S. mutans* by TPE-NIM^+^. White arrow indicates stained *S. mutans* and yellow arrows indicate non-stained *P. gingivalis*.

As shown in Figure 10, in different fields of vision, the rod-shaped *P. gingivalis* treated with the extract had no fluorescent signal, while the *Streptococcus*-shaped *S. mutans* had a fluorescent signal on the surface. It showed that TPE-NIM^+^ had a staining effect on *S. mutans* (G^+^ bacteria) but had no staining effect on *P. gingivalis*. AIEgens generally have no or weak fluorescence in the solution states but are strongly fluorescent in aggregate states owing to the restriction of intramolecular motions. The tertiary amine in TPE-NIM^+^ can interact strongly with lipoteichoic acid and butyoxylic acid present in the cell wall of G^+^ bacteria through electrostatic interaction, accumulate on the cell wall, and then emit fluorescence. As shown in Figure 11, we focused on comparing the staining difference between *S. mutans* (Figure 11A–C) and *P. gingivalis* (Figure 11D–F) and the TPE-NIM^+^ selective staining of G^+^ bacteria (*S. mutans*) in the bacterial mixture (Figure 11G–L). TPE-NIM^+^ successfully targeted and stained *S. mutans*, while no fluorescence was observed for *P. gingivalis*. These results further supported that TPE-NIM^+^ had a strong staining affinity for G^+^ bacteria.

**Figure 12:**
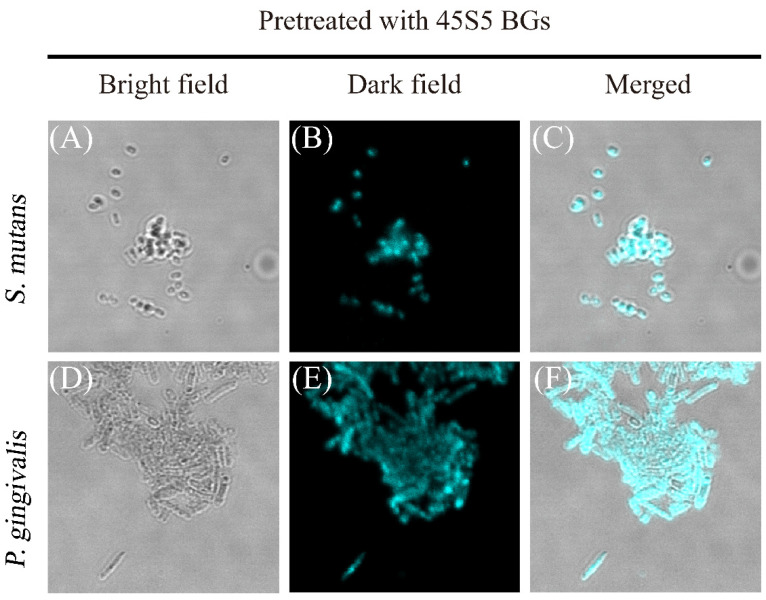
Confocal images of *S. mutans* (**A**–**C**) and *P. gingivalis* (**D**–**F**) after treatment with 45S5BGs and PLGA/10%BG/4%TPE-NIM^+^ membrane extract (with 45S5BGs), each for 15 min.

As shown in Figure 12, after 45S5BGs treatment, many *S. mutans* and *P. gingivalis* were killed, and the killed bacteria appeared as clumps of adhesion and aggregation. After the bacteria died, the integrity of the cell wall and cell membrane was destroyed. TPE-NIM^+^ not only accumulated on the cell wall of G^+^ bacteria but also entered the cells of both G^+^ and G^−^ bacteria to reunite. Therefore, whether it was G^+^ or G^−^ bacteria showing fluorescence was unclear. The fluorescence was not only reflected on the cell wall but on the whole bacterial body.

## 4. Discussion

In the remineralization experiment, the 45S5BGs in the membrane were degraded after a short time of soaking. Calcium and phosphorus ions, as important constituents of apatite-like structure, were evenly dispersed on the surface of the film, providing preliminary conditions for the formation of apatite-like structure which facilitated the binding of biologically active molecules in the body fluids through adsorption, aggregation, and chemical bonds. One study [36] has shown that the apatite-like structure could integrate with collagen fibers secreted by cells and form a strong chemical bond with newly-formed bone extending from the host bone. Therefore, as the 45S5BGs dissolved, calcium and phosphorus ions released to the membrane surface could promote the differentiation of bone marrow mesenchymal stem cells.

The GTR membrane is in direct contact with body fluids, blood, and so forth, therefore, during the application, a good hydrophilicity is one of the important influencing factors. Studies [20,37,38] showed that the surface of the material with a medium water contact angle in the range of 30–60° was conducive to the adsorption of serum proteins. The preferentially adsorbed albumin was exchanged by the cell adhesion serum proteins (such as fibronectin or vitronectin) and, ultimately, promoted cell adhesion. The composite membrane we prepared with the contact angle of about 40° is of great significance for clinical applications.

*P. gingivalis* (*P. g*) and *S. mutans* (*S. m*) were chosen in bacteria-related experiments; peri-implantitis and periodontitis are highly correlated with the chosen bacteria. The main pathogenic microorganisms of peri-implantitis are similar to periodontitis [39] and are importantly related to the attachment of subgingival plaques [40]. The plaque is mainly composed of G^−^ anaerobic bacteria, such as *P. g*, which uses a series of pathogenic factors, such as lipopolysaccharide, capsular polysaccharide, fimbriae, and gingival protease, to invade local periodontal tissues and escape host immune defenses [41]. It is one of the important pathogens that can induce periodontitis. The surface material of the cell wall of *S. m* promoted bacterial adhesion, aggregation, and plaque formation, which can induce the formation of subgingival plaques and provide favorable conditions for the growth and reproduction of subgingival bacteria [41]. Studies [8,17,18,19] confirmed that the antibacterial effect of 45S5BGs mainly due to the change in the pH value and the piercing effect of 45S5BG particles on bacteria. The normal form of the live bacteria in the liquid medium should be dispersed and free. A confocal laser microscope was used to observe the bacterial liquid after 45S5BGs treatment. Under the confocal microscope, we found that bacteria aggregated in the clumps and stained by the fluorescent probes, implying that TPE-NIM^+^ entered the cells of the dead bacteria and aggregated inside with the emission of strong fluorescence.

In addition to fluorescent dyeing properties, TPE-NIM^+^ also showed some antibacterial properties. Around 50–80% of the dry weight of bacteria is protein. The main component of the cell wall of bacteria is protein, which is composed of 20 amino acids connected by peptide bonds in a certain sequence. Therefore, the isoelectric point of bacteria is similar to that of amino acids. The pH value of Gram-positive bacteria is generally 2–3, and the pH value of G^−^ bacteria is generally 4–5. The process of culturing, staining, testing, and utilization of bacteria are all under alkaline (pH = 7–7.5), neutral, and acidic (pH = 6–7) conditions, which are all higher than the isoelectric point of bacteria. Therefore, bacteria are all negatively charged. This provides the reason why TPE-NIM^+^ had antibacterial properties. TPE-NIM^+^ had a positive charge. The electrostatic attraction/interaction between the positively charged amine and the negatively charged plasma membrane and the hydrophobic interaction between the TPE-NIM^+^ and the hydrophobic lipid tails might inhibit the respiration of the bacteria or change the number of surface charges, thus killing them [26].

In this study, AIE nanoparticles were innovatively added to the GTR membrane as biological probes which has important auxiliary function significance for the clinical diagnosis. These molecules with AIE characteristics aggregated into the nanoaggregates to emit fluorescence, so that pathogens can be detected in a simple, minimally invasive, fast, and wash-free protocol. After GTR surgery, the body fluid in the operation area could be extracted at any time without any elution or treatment. It could be directly observed under a fluorescence microscope to quickly diagnose whether it was infected and also discriminated G^+^ or G^−^ bacterial infection so that doctors could, in a timely fashion, prevent and control potential infections. 

At present, the GTR membrane is usually designed as a scaffold structure to promote better cell growth and better regeneration of periodontal tissue [36,42,43]. Therefore, we will foam or spin the membrane in further experiments to form a scaffold structure.

## 5. Conclusions

In summary, we successfully synthesized a novel PLGA/BG/TPE-NIM^+^ membrane which can be used in the GTR surgery and investigate its potential therapeutic effect for common oral bacterial infections. The membrane showed significant antibacterial properties due to its main antibacterial component—45S5BG. Meanwhile a nano material—TPE-NIM^+^—derived from our previous study, was used as a bioprobe in the membrane to assist clinical diagnosis in this study, and the results showed the nanoparticles also had certain antibacterial property. Besides, all components of the membrane have good bio-compatibility. Overall, this membrane can prevent post-GTR infections and help clinicians perform rapid, minimally invasive, and convenient clinical diagnoses for infections. Overall, this study provides a promising strategy to improve the antibacterial effect and bacterial tracer performance of GTR membrane.

## Data Availability

The data presented in this study are available on request from the corresponding author.
